# P02.52. Clinical trial of integrative medicine treatment approaches for migraine and headache disorders - a subgroup analysis of SIMTAP

**DOI:** 10.1186/1472-6882-12-S1-P108

**Published:** 2012-06-12

**Authors:** R Bonakdar, R Coeytaux, R Roberts

**Affiliations:** 1 Scripps Center for Integrative Medicine, La Jolla, USA; 2 Duke Clinical Research Institute, Durham, USA

## Purpose

As a component of the Study of Integrative Medicine Treatment Approaches to Chronic Pain (SIMTAP), we examined the utilization and efficacy of these approaches in a subset of patients with headache disorders. Specifically, we evaluated the ability to reduce headache disability and predictors of treatment response.

## Methods

The SIMTAP enrolled 142 subjects with headaches at 9 clinical sites between May, 2009 and October, 2010. A subset of these subjects (N=38), who suffered with headache disorders referred to one clinical site by a neurologist, were further analyzed. In addition to baseline demographics and treatment history, subjects had evaluation of hs-CRP, Vitamin D, BPI, and MIDAS at baseline, 6, 12 and 24 weeks.

## Results

At baseline, 55.2% had insufficient Vitamin D levels (<30 ng/mL) and 68.4% had an hsCRP level >1 mg/l. Mean baseline MIDAS score was 46 (SD=43) with 89% categorized as III or IV, meeting criteria for moderate to severe disability. The most common treatments attempted were biofeedback, acupuncture and manual therapy. 44.7% and 31.6% of subjects were able to reduce their MIDAS level by one or two categories. BMI, hsCRP or serum Vitamin D level at baseline did not predict improvement in headache disability.

## Conclusion

Headache sufferers evaluated at an integrative medicine facility appear to have a significant burden of disease based on baseline MIDAS scores, as well as characteristics which may be related to their headaches, including elevated CRP and insufficient Vitamin D levels, which did not appear to predict improvement in this preliminary analysis. Individualized integrative medicine treatment approaches were not associated with significant decreases in mean MIDAS scores at 12 and 24 weeks, but 44.7% of subjects reduced their headache burden by at least one category. Further research is needed to better understand clinical response to integrative medicine approaches for headache and identifying predictors of response.

